# Research progress into the principles and methods underlying capsular typing of *Glaesserella parasuis*

**DOI:** 10.1186/s13567-024-01395-7

**Published:** 2024-10-15

**Authors:** Yaxin Zhu, Lijun Guan, Junfeng Zhang, Yun Xue, Zhanqin Zhao

**Affiliations:** 1 Key Lab of Animal Bacterial Infectious Disease Prevention and Control Technology, College of Animal Science and Technology, Henan University of Science and Technology, Luoyang, 471003 China; 2 Key-Disciplines Lab of Safety of Environment and Animal Product, College of Animal Science and Technology, Henan University of Science and Technology, Luoyang, 471003 China

**Keywords:** *Glaesserella parasuis*, capsule, serotype, gel immunodiffusion, indirect hemagglutination assay, polymerase chain reaction

## Abstract

*Glaesserella parasuis* (GPS) is an important bacterial pathogen of swine. Serotype identification has presented a bottleneck in GPS research since it was first identified as the pathogen causing Glässer’s disease in pigs in 1910. This paper presents a systematic review of the history of the development and application of gel immunodiffusion (GID), indirect hemagglutination assay (IHA), and polymerase chain reaction (PCR) typing methods for GPS, and the discovery of their shared antigenic basis. It provides a systematic theoretical overview of the immunology and principles underlying the three typing methods and offers new ideas for research into the prevention and control of Glässer’s disease. In 1992, GPS was first classified into serotypes 1–15 using GID based on GPS heat-stable antigens, but about 25% of the strains were found to be non-typeable, and the composition of their antigens for serotyping was unclear. In 2003, the IHA method was established based on saline-extracted antigens of GPS, whose sensitivity and typing rate were higher than for GID, although about 15% of strains were still found to be non-typeable. The results of IHA and GID typing are roughly consistent, since they share the same GPS surface polysaccharide serotyping antigens, although whether these are capsular polysaccharides, lipopolysaccharides, or other polysaccharides, remains to be determined. In 2013, the Capsular polysaccharide (CPS) synthetic gene clusters from GPS serotypes 1–15 were successfully analyzed, confirming that CPS is essential for the formation of antigens for serotyping. In 2015, primers were designed based on the specific target genes of GPS capsules to establish a PCR typing method (H-PCR) for GPS, which, however, could not identify serotypes 5 and 12. In 2017, a new PCR typing method (J-PCR) was established based on the specific target genes of GPS capsules, which could identify serotypes 5 and 12. A combination of the two PCR typing methods enables the typing of almost all GPS strains, and the consistency with GID and IHA was verified using molecular biological methods. The antigenic basis of the three typing methods was shown to involve the GPS capsule. PCR typing methods are characterized by simple operation, fast speed, and low cost, and can successfully solve many problems in GID and IHA serotyping, and so have become widely adopted.

## Introduction

*Glaesserella parasuis* (GPS) is a Gram-negative bacterium in the Pasteurellaceae family that causes Glässer’s disease in pigs, by polyserositis, arthritis, and sepsis [1]. GPS has become a widespread worldwide pathogen in recent years, and is an important bacterial infectious disease in the swine industry [2, 3]. While pigs of all ages can become infected, weaned and nursery piglets are especially susceptible [4]. At present, vaccination is the major preventive measure for Glässer’s disease, and many inactivated vaccines are widely available commercially. However, due to the many GPS serotypes and the lack of cross-protection against them, the protective effect of vaccines varies [5–7].

Serotypes are microbial surface-specific antigenic types classified on the basis of serum antibody responses, and these antigens are usually important surface polysaccharides or proteins, including capsules, lipopolysaccharides (LPS), flagella, and pili. Serotyping is an important means of identifying the structural components and antigenic characteristics of pathogenic microbes, as well as an important tool in infectious disease surveillance, pathogen phylogenetic research, and the development of diagnostic reagents and vaccines [8–10]. The traditional methods of GPS serotyping include gel immunodiffusion (GID) and indirect hemagglutination assay (IHA), both of which are based on the reactions between antisera and bacterial surface antigens. GPS has been classified into 15 serotypes using GID and IHA, but a considerable number of strains remain non-typeable [11, 12]. The antigenic component for GPS serotyping in the two methods has long been unclear, and while it has been speculated to be a polysaccharide, it is not known whether it is a capsular polysaccharide (CPS) or an LPS. Howell et al. analyzed this component in reference strains of GPS serotypes 1–15 and found that the region contained *wza*, *wzb* and *wzc* homologues encoding a class I capsular loci. They therefore concluded that this region actually encoded for a CPS, that the GPS capsule was a class I type, and that the type of capsular locus was highly correlated with the serotype of the isolate and was a primary determinant of the GPS serotype [7]. Howell et al. established a polymerase chain reaction (PCR) typing method based on the capsule-specific genes of 15 reference serotype strains, and found that this method was faster and more accurate than the GID and IHA methods, with a higher typing rate [13]. In this paper, we systematically review the history of the development and application of the GID, IHA, and PCR typing methods for GPS, as well as the discovery of their shared antigenic basis, to provide a theoretical overview of the immunological basis and principles of the three typing methods, and offer new ideas for research and development into the prevention and control of Glässer’s disease.

## GID typing

In 1952, four different serotypes of GPS (A, B, C, and D) from 120 GPS isolates using precipitation tests with 37 °C surface extracts of GPS as antigens were identified [14]. Morozumi and Nicolet detected 32 GPS isolates from Japan and Switzerland using agglutination and GID tests with untreated somatic antigens and somatic supernatants treated at different temperatures (4 °C, 60 °C, 100 °C, and 121 °C) as antigens. The results showed that 26 isolates could be specifically identified as belonging to five different serotypes (serotypes 1–5) based on heat-stable antigens treated under high pressure at 121 °C. In the same year, they further identified serotypes 6 and 7 using the same method [11, 15]. This specific antigen was heat-stable, soluble, and not susceptible to pronase treatment, but could be extracted using phenol, suggesting that its main component could be a polysaccharide. Kielstein et al. identified 158 GPS isolates from Germany (including eight indole-positive GPS-like strains) using GID tests and the method established by Morozumi and Nicolet using the autoclaved somatic antigen of GPS. One hundred and fifteen strains (115/158, 72.8%) were successfully typed, of which 95 (95/158, 60.1%) were identified as the known serotypes 1–5 ([11]), and the remaining 20 (20/158, 12.7%) as seven novel serotypes (Jena 6–12). However, the five strains Jena 7–9 were later judged not to be of GPS since they were positive for indole, and were therefore eliminated [16]. Rapp-Gabrielson et al. detected 243 GPS isolates from North America using GID tests with the heat-stable autoclaved somatic antigen of GPS. A total of 14 serotypes were identified, nine of which were the known serotypes C, D, 1–7, and five of which were new serotypes (ND1–ND5) [17]. By this point, serotypes A–D, 1–7, Jena 6–12, and ND1–ND5 had been identified by GPS serotyping in independent studies by various scholars from different countries.

In 1992, GID tests were carried out using the specific rabbit antiserum and heat-stable antigen to further integrate and innovate the above GPS serotyping research results [12]. The previously defined serotypes 1–7 were preserved, while Jena and ND were designated as serotypes 8–15, thus rearranging the previously identified serotypes. GPS was now therefore classified into 15 serotypes (Table 1), with 15 identified reference strains, establishing the standard scheme for GPS serotyping (the Kielstein-Rapp-Gabrielson scheme, KRG) that is still in use today [12]. The study serotyped 214 isolates from Germany, in which 214 (214/290, 73.8%) strains were successfully serotyped, and the remaining 76 strains (76/290, 26.2%) could not be typed using this method (Table 4).

**Table 1:** **Proposed reference strains for 15 serovars of *Glaesserella parasuis*** [12]

Serovar	Reference strain	Country or origin	Diagnosis/isolation site	Previous serovar designation (s)	References
1	No.4	Japan	Healthy/nose	1	[11]
2	SW140	Japan	Healthy/nose	2, A	[11]
3	SW114	Japan	Healthy/nose	3	[11]
4	SW124	Japan	Healthy/nose	4	[11]
5	Nagasaki	Japan	Septicemia/meninges	5, B	[11]
6	131	Switzerland	Healthy/nose	6	[11]
7	174	Switzerland	Healthy/nose	7	[11]
8	C5	Sweden	Unknown	C	[11]
9	D74	Sweden	Unknown	D, Jena12	[11, 16]
10	H555	Germany	Healthy/nose	Jena10	[16]
11	H465	Germany	Pneumonia/trachea	Jena11, ND2	[12, 16]
12	H425	Germany	Polyserositis/lung	Jena6, ND5	[12, 16]
13	84–17975	United States	Unknown/lung	ND4	[12]
14	84–22113	United States	Unknown/joint	ND3	[12]
15	84–15995	United States	Pneumonia/lung	ND1	[12]

With the establishment of the KRG scheme, the serotypes of GPS have been better clarified and a complete GID method for GPS serotyping was established, and this has become an important and widely used technique in GPS serotyping. For example, Blackall et al*.* serotyped 31 GPS isolates from Australia according to the KRG scheme using GID tests with the autoclaved somatic antigens, and successfully typed 26 strains (26/31, 83.8%) (Table 4). Using this method, the cross-reaction between serotypes 7 and 10 was found for the first time [18]. Rafiee and Blackall prepared antisera against 15 reference strains in the KRG scheme by autoclaving, the 15 reference strains in the KRG scheme were then tested using the GID test with the 15 kinds of antisera prepared. Fourteen kinds of specific antisera were obtained, antiserum type 4 being the exception, that underwent one-way cross-reaction with the type 14 antigen. However, they believed that the preparation of high-titer antisera in rabbits in the KRG scheme presented difficulties since four or more rabbit immunizations were required for each antigen serotype, and that, even then, the preparation sometimes failed, requiring new rabbits or new reference strains (types 1 and 7) for repeat preparations [19]. Luppi et al*.* serotyped 106 GPS isolates from northern Italy using the GID test with hyperimmune rabbit antisera and autoclaved somatic antigens, in which 77 (77/106, 72.7%) strains were successfully serotyped and the remaining 29 (29/106, 27.3%) were non-typeable [20].

The GID typing method for the serotyping of GPS in the KRG scheme requires simple equipment and can be routinely performed in most laboratories. It is widely performed worldwide and has become an internationally recognized serotyping method [11, 12, 19]. However, about 30.6% of the strains investigated were still non-typeable by this method, because of the difficulty of antiserum preparation and the occurrence of cross-reactions (Table 4) [19]. More importantly, the antigenic substance for GPS serotyping using the GID method remained unclear. It was speculated to be a polysaccharide, but its specific composition requires further study even today [12].

## IHA typing

The KRG scheme has identified 15 GPS serotypes and is an internationally recognized serotyping method. However, some serotyping studies have shown that some GPS isolates cannot be typed using the KRG scheme, thus demonstrating its limitations [12, 19]. In addition, because the antigenic components for GPS serotyping remain unclear, other serotyping methods have been developed. In IHA tests, soluble antigens (generally polysaccharides) are first adsorbed onto the surface of granular carriers unrelated to immunity (such as red blood cells), and the resulting sensitized carriers are then bound to corresponding antibodies to connect and aggregate the red blood cells, resulting in agglutination reactions. IHA has been applied to the serotyping of *Actinobacillus pleuropneumoniae* [21, 22]. Del Río et al. prepared 15 serotype reference antisera for GPS, and serotyped GPS using IHA and sheep red blood cells (SRBC) as carriers and either “saline extract”, “boiled extract” or “autoclaved extract” as antigens [23]. The results showed that the antigens for serotyping adsorbed on SRBC are specific, and that saline extract exhibits the highest specificity and sensitivity, followed by boiled extract. Although autoclaved extracts are the preferred antigen in GID [11, 19], they exhibit the lowest serotype specificity in IHA, probably because heating for two hours at 121 °C alters the immunological activity of the target antigen, thus reducing or removing the adsorption capacity of SRBC. The use of saline extract without heat treatment has the highest specificity in serological reactions, suggesting that certain GPS surface antigens, not deep antigens, are associated with the specificity of antigens for serotyping, as in the case of the serotyping of *A. pleuropneumoniae* [24]. Finally, Del Río et al. serotyped 67 strains using IHA and GID with the saline extract of GPS as antigens, giving typing rates of 91.0% (61/67) and 62.7% (42/67), respectively, suggesting that IHA has a significantly higher GPS typing rate than GID (Table 4) [23].

Tadjine et al. prepared rabbit antisera for 15 reference strains and serotyped 300 GPS isolates, with the boiled extracts of autoclaved bacterial solutions of the 15 reference strain antigens as common antigens for GID and IHA. The results showed that cross-reactions were frequent and that more than 30% of the strains could not be typed using GID, while no cross-reactions occurred using IHA and the typing rate was greater than 90% (Table 4) [25]. Bacterial LPS or capsule can be adsorbed directly onto the surface of SRBC, while protein antigens can only be adsorbed following pretreatment of red blood cells with tannic acid, diaminobenzidine, and chromium chloride [26]. Therefore, Tadjine et al. speculated that the heat-stable and serotype-specific antigens adsorbed on the surface of SRBC in the boiled extracts might be LPS (or capsule) rather than protein antigens. In addition, high concentrations of antigen and antibody were required to generate visible precipitation reactions using GID based on the agar reaction system, thus demonstrating low sensitivity, while antigens and antibodies bound directly to each other without the need for a medium using IHA, thus demonstrating high sensitivity. Therefore, Tadjine et al. argued that the sensitivity of IHA is far higher than that of GID in GPS serotyping [25]. Angen et al. successfully typed 30 out of 57 clinical isolates (30/57, 52.6%) using GID with autoclaved extracts as antigens, and successfully typed 49 clinical isolates (49/57, 85.9%) using IHA with boiled extracts as antigens (Table 4) [23]. The typing results of GID and IHA were therefore roughly consistent, and slight deviations were only found in three isolates, although the typing rate of IHA was much higher than GID [27]. Cai et al. successfully typed 178 out of 281 GPS isolates (178/281, 63.3%) using GID with the autoclaved extracts as antigens, and successfully typed 254 isolates (254/281, 90.4%) using IHA with the saline extracts as antigens. Similarly, it was shown that the typing results obtained using GID and IHA were roughly consistent, but that the typing rate using IHA was much higher than that using GID [28]. Zhang et al. first carried out serotype identification using GID with autoclaved extracts as antigens, and then performed IHA using the saline extracts as antigens. Eighty-nine of 112 clinical isolates were successfully typed, with a typing rate of 79.5%, and the remaining 23 strains could not be typed using either method [29].

In summary, the immunological principles and clinical applications of GID and IHA show that the two serotyping methods should share the same target antigen, but that IHA is more sensitive with a higher typing rate than GID (Table 4) [25]. However, even when GID and IHA were combined, a considerable proportion of strains could not be typed [27]. More importantly, the antigenic component for GPS serotyping remained unclear based on the studies carried out before Howell’s analysis of the GPS capsular gene cluster in 2013 [7]. It was thought to be a polysaccharide component, but whether it was a CPS or LPS remained in doubt. A number of problems persist regarding the use of GID and IHA for GPS serotyping: (1) the specific antisera are complicated to prepare and cumbersome to use; (2) the non-standardized antisera have different titers and their sensitivity often varies; (3) cross-reactions often occur in the serotyping of clinical isolates; and (4) the results need to be visually interpreted by the analysts, so that human error will always reduce the accuracy of typing [13].

## Molecular biological basis of GPS capsular typing

The capsule is a viscous layer that surrounds the cell wall of some bacteria and consists of glycosaminoglycan (GAG; formerly known as mucopolysaccharide, aminopolysaccharide, or acidic polysaccharide). It is a crucial structural component of bacteria and a pathogenic factor and plays a key role in adhesion to host cells, resistance to phagocytosis, and immune escape [30–33]. Based on the reaction between serum antibodies and surface antigens, GPS has been classified into 15 serotypes using GID and IHA. While the antigen for serotyping is thought to be a polysaccharide, it is not known whether it is a CPS, LPS, or a different polysaccharide [12, 25]. PCR typing methods based on genes associated with the biosynthesis of bacterial surface polysaccharides such as CPS or LPS, have been successfully used to serotype a wide variety of bacteria [34–37]. CPS or LPS play a vital role in the interaction between GPS and its host, and a clear identification of the polysaccharide type is key to the analysis of antigenic components for GPS serotyping.

Williamson et al. isolated the GPS capsular substance for the first time and found that it consisted of α-galactose-α-N-acetylglucosamine repeating disaccharide units linked by phosphodiester bonds [38]. Morozumi et al. confirmed the presence of capsules in nine clinical isolates of GPS by morphological observation (iridescence in colony biofilms and capsule staining), the acridine yellow agglutination test, and Cetavlon electrophoresis, and verified that the GPS capsules were composed of acidic polysaccharides and characterized by their solubility, heat resistance, and protease hydrolysis resistance [39]. Xu et al. first reported the whole genome sequence of GPS serotype 5 strain SH0165 (accession number: CP001321), and found a 14 kb polysaccharide biosynthetic gene cluster (*HAPS0039*–*HAPS0052*), which was annotated to encode O-antigen polysaccharides [7, 40].

**Figure 1:**
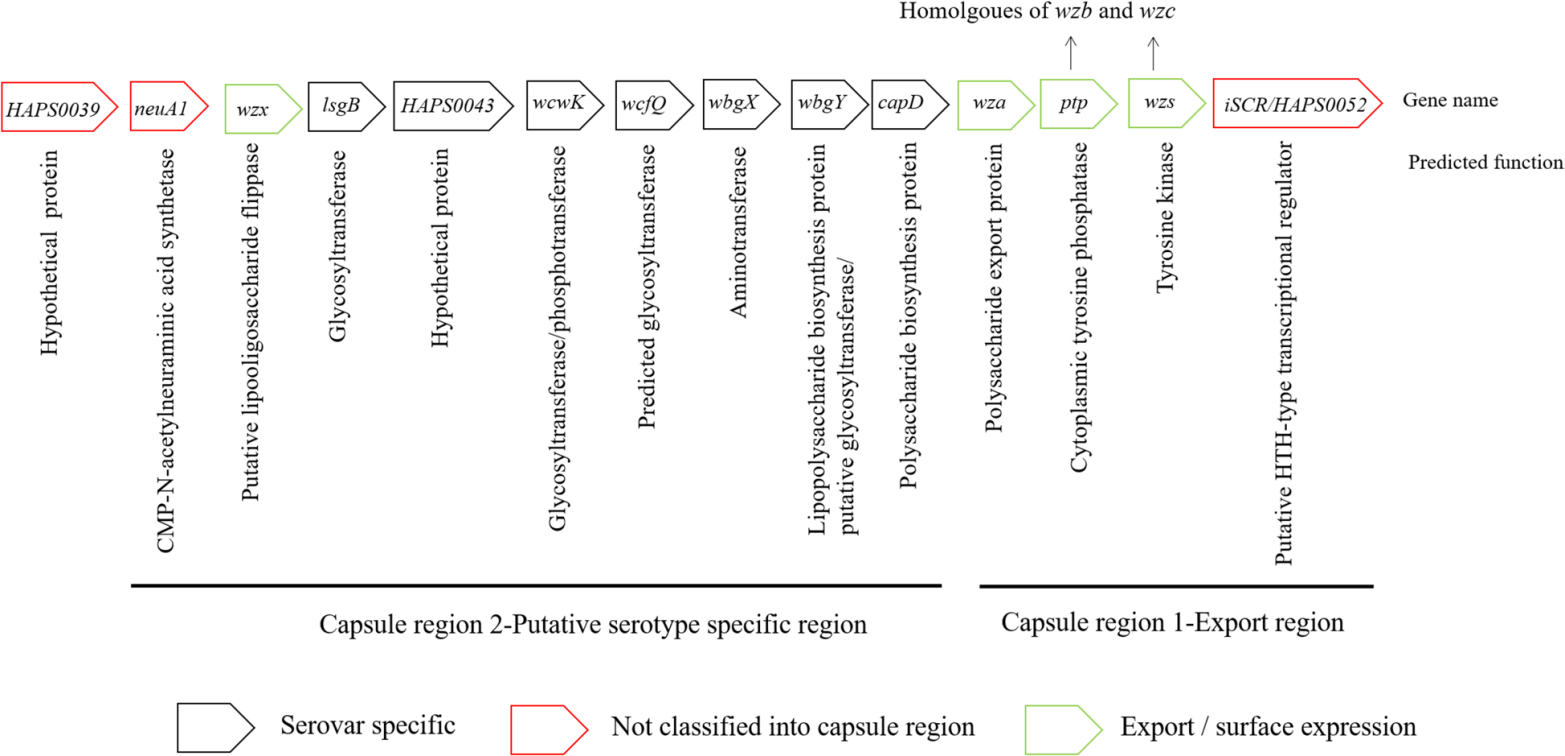
**Separation of the serovar 5 capsule locus into potential capsule regions, showing the details of their predicted functions, based on the published strain SH0165** [7].

**Figure 2:**
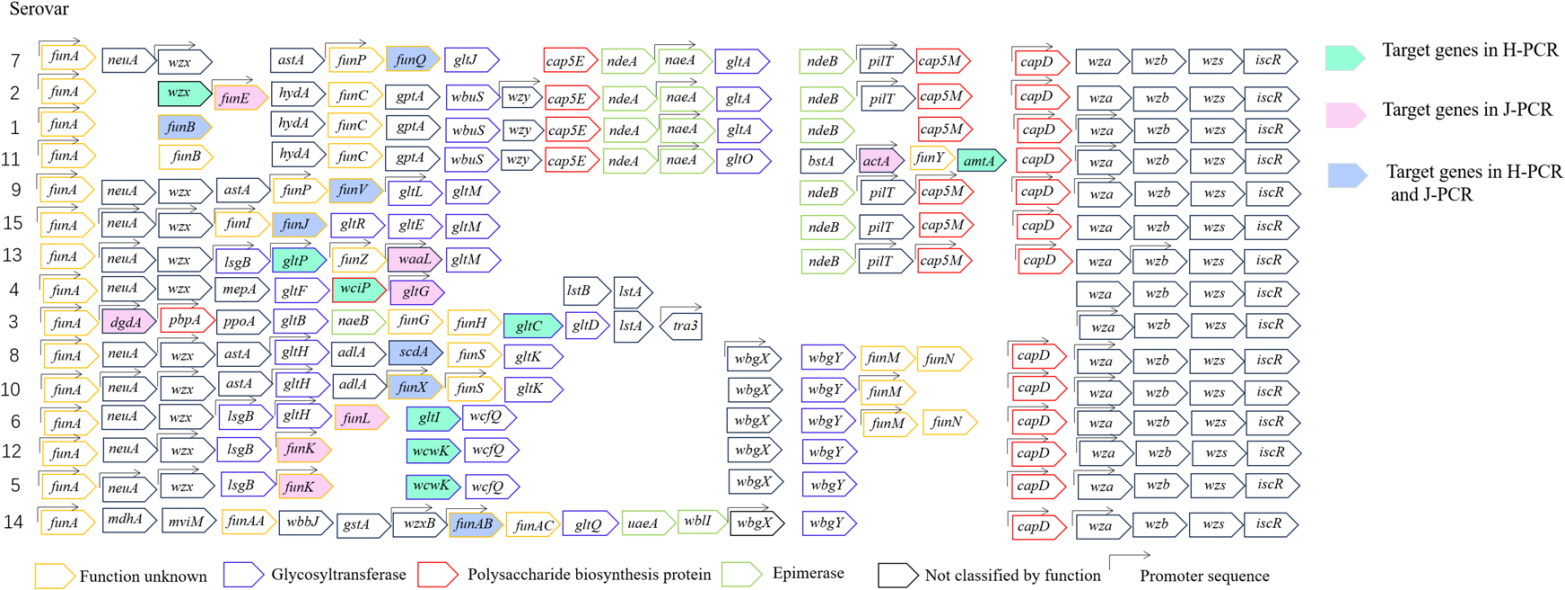
**Scheme of each capsule locus for all 15 reference strains of *Glaesserella parasuis*** [7].

Howell et al*.* conducted genomic sequencing on 15 serotype reference strains of GPS and compared their sequences with those of the gene cluster (*HAPS0039*–*HAPS0052*) of SH0165 [7]. It was found that this gene cluster was split into R1 and R2 regions, in which the R1 region (*HAPS0049*–*HAPS0051*) was a homologue of the *wza*–*wzc* gene and a unique conserved gene for class I capsule synthesis and export. The results confirmed that this gene cluster encoded the class I capsule of GPS rather than O-antigen polysaccharides (Figure 1) [41, 42]. *Wzx* and *wzy* were involved in the biosynthesis of both class I CPS and LPS or lipo-oligosaccharides (LOS), whereas *wza*-*wzc* was implicated only in the biosynthesis of class I CPS [42–45]. The gene clusters and functions of group I capsule biosynthesis are shown in Table 2 [42, 45]. The *HAPS0052* gene of GPS was located behind the R1 region and was a homologue of the *iscR* gene, and contained a helix-turn-helix DNA binding motif where it bound to the promoter region of the CPS synthetic gene cluster, which positively regulated the transcription of the capsular GAG gene (Figures 1 and 2). The *HAPS0039* gene was located in front of the R2 region, named *funA* (function unknown gene A) since it was the first gene with unknown functions discovered by Howell et al. There were also some genes with unknown functions in the R2 region, named *funB*–*funZ* (Figure 2). The R2 region was located between *funA* and *wza*, in which 13 serotype reference strains had different gene compositions, except serotypes 5 and 12, and included various genes involved in oligosaccharide repeat unit synthesis, which could synthesize CPS of different serotypes. Therefore, the R2 region was a potential serotype specific region and a target region for designing PCR typing primers (Figure 2) [7].

**Table 2:** **Genes involved in capsule biosynthesis** [42, 45]

Protein	Member of family	Location	Function (or putative function)
*wzx*	PST–1	Inner membrane (integral)	Transfers nascent undecaprenyl diphosphate–linked repeat units across the inner membrane
*wzy*	–	Inner membrane (integral) with periplasmic catalytic site	Putative polymerase; assembles undecaprenyl diphosphate–linked polymers using lipid–linked repeat units exported by *wzx*
*wzc*	MPA–1 (PCP–2a)	Inner membrane (integral) with a large periplasmic domain and cytosolic N and C termini	Participates in high–level polymerization of capsular polysaccharide and forms part of a *trans*–envelope capsule translocation complex; *wzc* activity is determined by cycling of its phosphorylation state via the cytosolic C–terminal tyrosine autokinase domain
*wzb*	PTP	Cytoplasm	Protein tyrosine phosphatase; dephosphorylates *wzc*
*wza*	OMA	Outer membrane	Forms a multimeric putative translocation channel and interacts with the periplasmic domain of *wzc*
*wzi*	heat–modifiable monomeric β–barrel protein	Outer membrane	It is a group I capsule–specific gene; the polymer is tightly bound to the cell surface with discrete capsule structure; adjust surface correlation
*wbap*	Galactose–1–P transferase belongs to PHTP protein family (member of polyisopentene phosphate hexose–1–phosphate transferase family)	Inner membrane	Initiation of O antigen and capsular biosynthesis in many bacteria by transfer of Gal–1–P or Glc–1–P to und–p (undecapentene phosphate carrier sugar receptor)

The CPS biosynthetic gene cluster of GPS serotypes 5 and 12 consisted of the same genes. However, a comparative analysis of the encoded amino acid sequence revealed 48 different amino acid sites in the remaining 12 coding sequences. Except for *iscR* and *wzb* in the R2 region, the most different sites were found in the genes *wbgY*, encoding for glycosyltransferase, and *wzx*, encoding for flippase, two of the key enzymes in CPS synthesis. Compared with serotype 12, the CPS biosynthetic gene cluster of serotype 5 contained an additional promoter [7], so that CPS of different serotypes could be synthesized. Moreover, the whole genomes of the reference strains of serotypes 5 and 12 were also significantly different, and the nucleotide sequence similarity between the two was only 81.7% [7].

Howell et al. compared the similarity of the capsular gene cluster sequences (*funA*–*iscR*) among 117 GPS strains, and found that the position and composition of the capsular gene clusters were the same among the GPS strains of the same serotype, even though their relationships were distant, indicating that the capsular gene cluster was the major determinant of the GPS serotype. They also found that the similarity of the capsular gene cluster sequence of serotype 6 was above 85%, while it was more than 98% for serotypes 1, 2, 3, 4, 5/12, 7, 8, 9, 13, and 15 (only one strain was available for serotypes 10, 11, and 14, so their similarity could not be compared). In addition, 23 potential serotype-specific genes besides the capsular gene cluster were also identified in these strains, including five previously identified capsular genes, three phage genes, one transposase gene, one filamentous hemagglutinin gene, and 13 genes with unknown functions [46].

## PCR typing methods for GPS and their application

The successful analysis of the GPS CPS synthetic gene cluster in preliminary studies showed that this gene cluster determines the formation of GPS capsular serotypes [7, 46]. The R2 region of the GPS capsular gene cluster was a specific region for the synthesis of antigens for serotyping, including transaminase genes, glycosyltransferase genes, flippase genes, and some genes with unknown functions (Figure 2), whose similarity was less than 51% at the nucleotide level [7, 46]. Therefore, the PCR typing method for GPS capsular antigen could be established based on type-specific genes in the GPS CPS synthetic gene cluster.

Howell et al. designed primers targeting the specific gene sequences in the R2 region of the GPS CPS gene cluster and established a PCR typing method (H-PCR) for identifying GPS capsular serotypes 1–15. However, GPS serotypes 5 and 12 could not be distinguished using H-PCR due to their having the same gene composition as the CPS gene clusters [7, 13]. The GPS serotypes 1, 2 and 11 also could not be directly distinguished using H-PCR due to the lack of sufficient specific target genes or differential genes in the capsular gene clusters of the GPS serotypes 1, 2 and 11. In H-PCR, the specific target gene of primers selected for serotype 1 was *funB*, but serotype 11 contained the same *funB* gene, and serotype 2 contained the homologous gene *funE*, with high similarity. Hence, when the serotype 1 strain was identified using H-PCR, positive results emerged in serotype 11 and weakly positive results in serotype 2. Therefore, serotypes 11 and 2 need to be further differentiated from serotype 1 using their specific primers (target genes for serotypes 11 and 2: *amtA* and *wzx,* respectively). The target gene of primers selected for serotype 2 was *wzx*. Many other serotypes also contained the *wzx* gene, but serotype 2 had significantly different genetic variation sequences in the *wzx* gene, compared with the other serotypes. Using H-PCR, Howell et al*.* obtained a typing rate of 100% among 150 clinical isolates, and these results were completely consistent with those of capsular gene cluster sequencing-based typing (Table 4). IHA achieved a typing rate of 84.0% (98/117) among 117 strains, and the results were consistent with those of PCR typing among 84 strains, with a coincidence rate of 85.7% (excluding the non-typeable strains in IHA). The typing rate was also 100% among another 84 GPS strains using H-PCR (without undergoing capsular gene cluster sequencing-based typing), and was 77% (51/66) among 66 strains using IHA, the results being consistent with those of PCR typing among 45 strains, with a coincidence rate of 88.2% (excluding non-typeable strains in IHA) (Table 4). In summary, Howell et al.’s H-PCR method is rapid, sensitive, and specific. It further reduces the non-typeable rate, cross-reactions, and detection costs, and can detect crude DNA extracts directly derived from a bacterial colony [13], and has become widely used.

Ma et al. carried out serotyping on 100 GPS strains using PCR and GID simultaneously (mPCR). The typing rates of the two methods were 93.0% and 73.0%, respectively, with a coincidence rate of 87.7% (64/73), it should be noted that in this paper, when calculating the coincidence rate, strains that could not be typed by both methods were excluded. But, Ma et al.’s article included strains that could not be typed and showed a concordance rate of 66.0%. Capsular gene cluster sequencing was performed on 11 GPS strains, including nine strains with different typing results (excluding strains that could be typed by PCR but could not be typed by GID) and two strains that could not be typed by either PCR or GID [47]. Gene deletion, insertion, mutation, and drift were found in the capsular gene clusters of these strains, suggesting that genetic instability was present in the GPS capsular gene cluster, similarly to the findings in other bacteria [47–49]. Wang et al. achieved a typing rate of 92.1% (234/254) among 254 GPS isolates (from Sichuan, China) by using mPCR, but the remaining 20 strains could not be typed [50]. Nubia et al. obtained a typing rate of 93.3% (886/950) among 950 GPS isolates (223 from the United States, 436 from Europe, 167 from China, 68 from Canada, and 56 from Vietnam) using mPCR, but the remaining 64 strains could not be typed [51]. Silva et al. used mPCR for typing of 105 GPS isolates (from Brazil), with a typing rate of 100.0% [52]. In summary, the mPCR method established by Howell et al. can achieve an overall GPS typing rate of over 92%, although traditional GID or IHA is still required to distinguish serotypes 5 and 12 [7, 13].

Jia et al. performed a comparative analysis of the GPS CPS synthetic gene cluster sequences and found that serotype 12 contained a hypothetical gene with unclear functions. Serotype 5 and some other serotypes did not contain this hypothetical gene, and they designed primers for use with a new complete PCR typing method (J-PCR) to distinguish GPS serotypes 1–15 and identify serotypes 5 and 12 (Table 3) [53]. In J-PCR, the target genes of primers of serotypes 1, 7, 8, 9, 10, and 14 were the same as those used in H-PCR, but with different primer sequences. Both of the target genes of primers and primer sequences of other serotypes were different between J-PCR and H-PCR (Table 3) [53]. Jia et al. serotyped 298 GPS strains using J-PCR and IHA simultaneously. The typing rates of the two methods were 94.3% (281/298) and 76.5% (228/298), respectively, with a coincidence rate of 98.2% (224/228) [53], which was slightly lower than that of H-PCR (85.7% and 88.2%) [13]. J-PCR is usually used in combination with H-PCR, because serotypes 5 and 12 that cannot be distinguished by H-PCR can be identified by serotype 12-specific primers in J-PCR [13, 53]. Li et al*.* serotyped 148 GPS isolates (from eastern China) using H-PCR plus J-PCR, and achieved a typing rate of 97.3% (144/148), with four non-typeable strains [54].

**Table 3:** **Serotyping multiplex primers and estimated product sizes** [13, 53]

Serotype	Direction	Howell et al. [13]			Jia et al. [53]		
		Targeted gene	Sequence (5′to 3′)	Product size, bp	Targeted gene	Sequence (5′to 3′)	Product size, bp
1	F R	*funB*	CTGTGTATAATCTATCCCCGATCATCAGC GTCCAACAGAATTTGGACCAATTCCTG	180	*funB*	TGCATAAAAAATTTTTGAA TTATATATATTTTACATTTCTAAG	1245
2	F R	*wzx*	CTAACAAGTTAGGTATGGAGGGTTTTGGTG GGCACTGAATAAGGGATAATTGTACTG	295	*funE*	ATGGAAGAAAAAGAATATATC TTAAAGTTTTGATTTGTCAATG	1032
3	F R	*glyC*	CATGGTGTTTATCCTGACTTGGCTGT TCCACATGA6GGCCGCTTCTAATATACT	650	*dGIDA*	ATGACTAAAAAAATTTTAGTTACAG TTACTTAATACCTAAGCG	1068
4	F R	*wciP*	GGTTAAGAGGTAGAGCTAAGAATAGAGG CTTTCCACAACAGCTCTAGAAACC	320	*gltG*	ATGAATAATAAAGTCTCAATTATAA TTACATATGTTTTACAATTCC	753
5/12	F R	*wcwK*	CCACTGGATAGAGAGTGGCAGG CCATACATCTGAATTCCTAAGC	450	*funK*	ATGCCAATAGAGATAGC CCTGCCATATTATGA	560
6	F R	*gltI*	GATTCTGATGATTTTTGGCTGACGGAACG CCTATTCTGTCTATAAGCATAGACAGGAC	360	*funL*	ATGAGTATTTTTTTTCTAATTG TTCCCTGATCATTGTAGTAACC	443
7	F R	*funQ*	CTCCGATTTCATCTTTTCTATGTGG CGATAAACCATAACAATTCCTGGCAC	490	*funQ*	TAGTTGGTATATTATTTTCT AGAATGCATCTGTACCACTAAG	600
8	F R	*scdA*	GGAAGGGGATTACTACTACCTGAAAG CTCCATAGAACCTGCTGCTTGAG	650	*scdA*	CAGCAGGTTCTATGGAGTCA CACATTATAACTTTCTTT	350
9	F R	*funV*	AGCCACATCAATTTTAGCCTCATCA CCTTAAATAGCCTATGTCTGTACC	710	*funV*	GCTCCAATATCAGCAGTA AGAGTAATGAGCATCTCCG	819
10	F R	*funX*	GGTGACATTTATGGGCGAGTAAGTC GCACTGTCATCAATAACAATCTTAAGACG	790	*funX*	TGATTATTCTACTGCCTTTA CACCTAGCGTAACCCATA	320
11	F R	*amtA*	CCATCTCTTTAACTAATGGGACTG GGACGCCAAGGAGTATTATCAAATG	890	*actA*	ATGATTATAGGTATTTATGGTGC CTATTTATTTTTTGAAAATTCTC	657
12	F R	–	–	–	*Hypothetical gene*	ATGGCTCACGATCCGAAAG ATTTCCCTTTCCTAAACGC	508
13	F R	*gltP*	GCTGGAGGAGTTGAAAGAGTTGTTAC CAATCAAATGAAACAACAGGAAGC	840	*waaL*	GGCATTAGAGTTTCACCTA TATTAGCATACCCAGCAT	800
14	F R	*funAB*	GCTGGTTATGACTATTTCTTTCGCG GCTCCCAAGATTAAACCACAAGCAAG	730	*funAB*	TGTCTTTGTTACTACTAATTATTG TAGTAACTCCAGATAAAGC	906
15	F R	*funI*	CAAGTTCGGATTGGGAGCATATATC CCTATATCATTTGTTGGATGTACG	550	*funJ*	TTCGCAAGTATAAGGGACT GATGTAGCCATAAAGTCAAT	536
All	F R	*HPS_219690793*	ACAACCTGCAAGTACTTATCGGGAT TAGCCTCCTGTCTGATATTCCCACG	275	–	–	–

Schuwerk et al*.* successfully serotyped 297 out of 308 GPS strains (from Germany) using H-PCR, with a typing rate of 96.4% (Table 4) [55]. Moreover, 46 strains identified as serotypes 5/12 were further distinguished using J-PCR into 16 strains of serotype 5, and 28 strains of serotype 12, as well as two non-typeable strains. However, when using the primers for serotypes 5/12, among the 28 strains identified as serotype 12 using J-PCR, only 10 strains were PCR positive, the remaining 18 strains being PCR negative, suggesting that Jia et al.’s universal primers for serotypes 5/12 were prone to false negative results. Therefore, the universal primers for serotypes 5/12 designed by Howell et al. based on the target gene *wcwK* can be used for GPS PCR typing. Among the other 251 serotyped strains, only 20 strains were inconsistent in the serotyping results of H-PCR and J-PCR, and were restricted to serotypes 1, 2, and 11. Specifically, 16 strains were identified by H-PCR as serotype 2 (target gene of primer: *wzx*), although weakly positive bands also emerged when primers for serotype 1 (target gene: *funB*) were used for identification, since the serotype 2 capsular gene cluster also contained the *funB* homologous gene *funE* [13]. However, these 16 strains were all specifically identified using J-PCR as serotype 2 since their primers originated from variant sequences completely different from *funB* in *funE*. Therefore, the primers designed by Jia et al. based on the target gene *funE* can be selected preferentially for identification of GPS serotype 2. Two other strains were identified as serotype 11 by both H-PCR and J-PCR (target genes of primers: *amtA* and *actA*), and positive bands also emerged when primers for serotype 1 (target gene: *funB*) were used for identification, since the serotype 11 capsular gene cluster also contained *funB* [13, 53]. Another two strains could not be serotyped by the two PCR methods, which were positive for serotypes 1, 2, and 11 using H-PCR, and positive for serotypes 2 and 11 using J-PCR. As mentioned above, gene deletion, insertion, mutation, and drift may have occurred in their capsular gene clusters, indicating genetic instability. In addition, many scholars have used these two PCR typing methods to serotype large numbers of GPS strains, achieving good serotyping results and demonstrating their suitability for wide use [55–59].

**Table 4:** **Comparison of three typing methods of GID, IHA, and PCR for GPS**

Year	Country or region	Number of strains	Typing rates and concordance rates of different typing methods				References
			GID	IHA	PCR	Concordance rate (%)^*^	
1992	Germany	290	73.8% (224/290)				[12]
1996	Australia	31	83.8% (26/31)				[18]
2000	Australia	46	58.8% (27/46)				[19]
2013	Northern Italy	106	72.7% (77/106)				[20]
2003	Spain	67	62.7% (42/67)	91.0% (61/67)		97.6	[23]
2004	North America	300	70.0% (210/300)	90.0% (270/300)			[25]
2004	Denmark	57	52.6% (30/57)	85.9% (49/57)		93.3	[27]
2005	China	281	63.3% (178/281)	90.4% (254/281)		94.4	[28]
2015	England, Wales, Denmark, Spain, Australia	234		81.4% (149/183)	100.0% (234 /234) ^H−PCR^	86.6	[13]
2016	China	100	73.0% (73/100)		93.0% (93/100) ^H−PCR^	87.7	[47]
2017	China	254			92.1% (234/254) ^H−PCR^		[50]
2021	America, Europe, China, Vietnam, Canada	950			93.3% (886/950) ^H−PCR^		[51]
2022	Brazil	105			100.0% (105/105) ^H−PCR^		[52]
2017	China	298		76.5% (228/298)	94.3% (281/298) ^J−PCR^	98.2	[53]
2018	China	148			97.3% (144/148) H-PCR + J-PCR		[54]
2020	Germany	308			96.4% (297/308) H-PCR + J-PCR		[55]
Total no. of strains typed by GID		1278	69.4% (887/1278)				
Total no. of strains typed by IHA		1186		85.2% (1011/1186)			
Total no. of strains typed by PCR		2397			94.8% (2274/2397)		

H-PCR + J-PCR, H-PCR and J-PCR were used in conjunction.

^*^In this paper, when calculating the coincidence rate, strains that could not be typed by both methods were excluded.

## Summary and prospects

Glässer’s disease is one of the most important bacterial infectious diseases threatening the swine industry worldwide, and GPS serotyping is a key to understanding Glässer’s disease epidemics and achieving their prevention and control [4]. The GPS serotyping method (serotypes 1–15) established by Kielstein and Rapp-Gabrielson in 1992 was the first use of a GID test using heat-stable antigens but failed to identify about 25% of the strains, and the composition of their antigens for serotyping remains unclear [12]. The GPS IHA method, established by Del Río et al*.* based on saline-extracted antigens in 2003, was characterized by simple antigen preparation and higher sensitivity and typing rates than GID, and has gradually become the gold standard for GPS serotyping, and made a significant contribution to GPS serotyping [23]. The typing results were roughly consistent between IHA and GID, and use the same surface GPS polysaccharides as serotyping antigens, but whether these were CPS or LPS (or LOS), or even other polysaccharide substances, remains to be shown [12, 16, 23, 25].

The successful analysis of the CPS synthetic gene cluster from GPS serotypes 1–15 by Howell et al. in 2013 confirmed that this gene cluster is essential for the formation of the GPS capsular serotype [7]. Howell et al. designed primers based on the specific target genes of GPS capsules and established the H-PCR typing method for GPS, which, however, could not identify serotypes 5 and 12. They also verified the consistency between GID and IHA typing results, and proved that the GPS capsule is the antigenic basis of the GID, IHA, and PCR typing methods [13]. The similar J-PCR typing method established by Jia et al. in 2017, based on the specific target genes of GPS capsules, could identify serotypes 5 and 12 [53]. The combination of the two PCR typing methods enabled typing of almost all GPS strains, and is characterized by a simple operation, fast speed, and low cost, and successfully solves many problems in GID and IHA serotyping, such that the two methods have been widely applied in combination [55–59].

As the history of the establishment and application of GPS typing methods shows, non-specific reactions often occur during GPS serotyping using GID based on heat-stable antigens [18, 19], presumably due to the interference of the heat-stable LPS (or LOS) antigen. CPS can be purified, its antigen titer can be calibrated, and the standard serum antibodies prepared from purified CPS can be used during GPS serotyping using GID, thus reducing or eliminating the interference of LPS (or LOS) and other antigens, and the occurrence of non-specific reactions. Similarly, non-specific reactions in IHA typing could also be attributed to LPS (or LOS) antigens, since they have similar characteristics in adsorbing SRBC to CPS [26]. Therefore, non-specific reactions can also be reduced or eliminated in this way. Future studies should further analyze the composition and structure, gene clusters and functions, and molecular synthesis mechanisms of GPS CPS to better understand the molecular immune response mechanism of CPS typing [60].

Regarding GPS typing using PCR methods, H-PCR can be used in conjunction with J-PCR. Combining J-PCR with H-PCR further achieves the identification of serotypes 5 and 12 [13, 53]. However, Jia et al.’s universal primers for serotypes 5 and 12 were prone to give false negative results. Therefore, the universal primers for serotypes 5 and 12, designed by Howell et al. based on the target gene *wcwK*, should be used first, followed by application of the primers for serotype 12 designed by Jia et al. [55]. Since non-specific positive bands of serotype 1 also emerge in serotype 2 strains in H-PCR, the primers designed by Jia et al., based on the target gene *funE*, should be preferentially selected for identification of GPS serotype 2 [13, 53]. In addition, GPS capsular gene clusters demonstrate some genetic instability, and gene deletion, insertion, mutation, and drift may occur, resulting in a small number of non-typeable strains, which can be further analyzed using DNA sequencing to discover their structural characteristics [47–49, 55].

CPS and LPS (or LOS) are two vital surface antigen substances for serotyping Gram-negative bacteria. Studies have shown that GPS, like other bacteria of the Pasteurellaceae family, contain LOS antigens as well as CPS antigens [60–65]. To better understand the basic structure and serotypes of GPS, it is necessary to systematically analyze the serotype, composition and structure, and biosynthetic gene clusters and functions of GPS LOS [60].

## Data Availability

The data and materials will be available upon request.

## Abbreviations

GPS: *Glaesserella parasui* *s*
GID: gel immunodiffusion
IHA: indirect hemagglutination assay
PCR: polymerase chain reaction
CPS: capsular polysaccharide
LPS: lipopolysaccharides
KRG: Kielstein-Rapp-Gabrielson scheme
SRBC: sheep red blood cells
GAG: glycosaminoglycan
LOS: lipo-oligosaccharides
*funA*: function unknown gene A
*funB*–*funZ*: function unknown gene B–function unknown gene Z
H-PCR: Howell et al.(2015)’s [13] PCR
J-PCR: Jia et al. (2017)’s [53]PCR
